# An Assessment of Cellular Atypia Utilizing Line‐Field Confocal Optical Coherence Tomography (LC‐OCT)

**DOI:** 10.1111/srt.70227

**Published:** 2025-09-12

**Authors:** Shazli Razi, Priya Agarwal, Gaurav N. Pathak, Babar K. Rao

**Affiliations:** ^1^ Department of Internal Medicine Hackensack Meridian Ocean University Medical Center Brick New Jersey USA; ^2^ Department of Internal Medicine Jersey Shore University Medical Center Neptune New Jersey USA; ^3^ Rutgers Robert Wood Johnson Medical School New Brunswick New Jersey USA; ^4^ Department of Dermatology Rao Dermatology Atlantic Highlands New Jersey USA; ^5^ Department of Dermatology Weill Cornell Medicine New York New York USA

**Keywords:** artificial intelligence, cellular atypia, keratinocytes, line-field confocal optical coherence tomography, skin cancer

1

To the Editor,

Line‐field confocal optical coherence tomography (LC‐OCT) is a non‐invasive and high‐resolution 3‐dimensional imaging modality that allows for real‐time visualization of the epidermis and upper dermis by generating horizontal and vertical images [1]. Artificial intelligence (AI) deep learning algorithms have been successful in analyzing LC‐OCT images and monitoring treatment progression, but have not been studied on a large scale [2, 3]. In this study, LC‐OCT images of three lesions were described and analyzed for atypia by an AI segmentation algorithm.

A previously validated deep learning model (DLM) consisting of healthy and pathologic keratinocytic samples from 185 3D LC‐OCT images and comprising greater than 3.7 million atypical or normal cells, was used for this study [2]. This model outperformed expert consensus by more than 20 points of area under the curve (AUC) score, receiving an AUC of 0.965 compared to individual experts' scores, ranging from 0.708 to 0.745, with a combined consensus of 0.766 [2]. Three pathologic samples of varying atypia were obtained from different patients. Metrics such as average atypia score, average epidermal thickness, stratum corneum thickness, dermal‐epidermal junction, and stratum corneum undulation were calculated using DLM. Atypia scores range from zero to one, with a score closer to one indicating a greater degree of atypia. Results are summarized in Figure 1.

**FIGURE 1:**
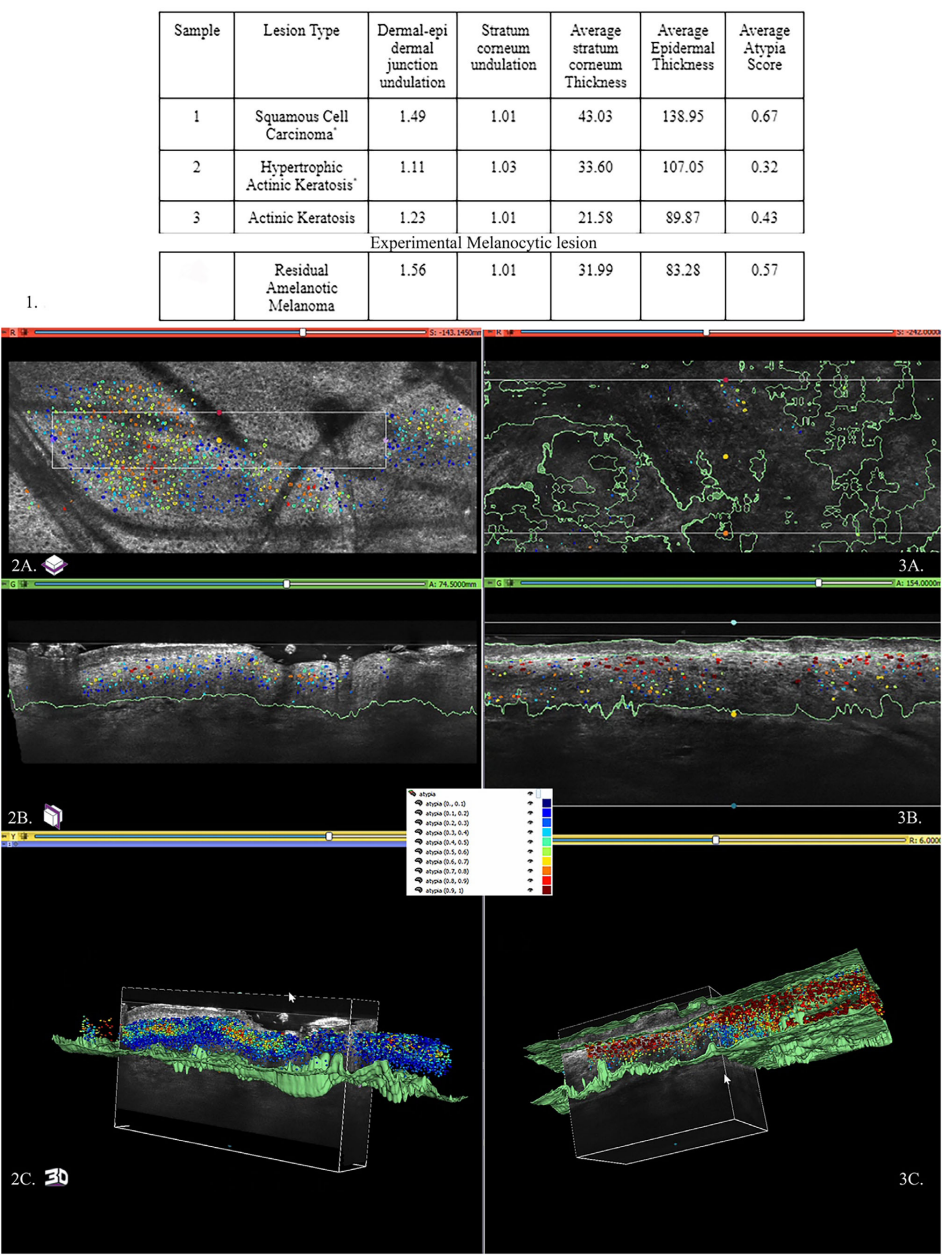
Atypia scores for the three samples are summarized, with sample 2 (2A–2C; lowest atypia score) shown on the left and sample 1 (3A–3C; highest atypia score) shown on the right. Lesion 1, 2, and the experimental amelanotic melanoma are biopsy‐proven.

As demonstrated by Figure 1, the highest atypia score generated by the DLM is seen in the squamous cell carcinoma (SCC) lesion as compared to the actinic keratosis (AK) and hypertrophic actinic keratosis (HAK) lesions. This is in line with the visual appearance of the LC‐OCT images, shown in Figure 1. Furthermore, as expected, the average epidermal thickness of the HAK lesion is higher than that of the AK.

The DLM has not yet been trained on the segmentation of melanotic lesions, and we have included the assessment of a residual amelanotic melanoma experimentally to evaluate the DLM's performance. Notably, it resulted in a higher atypia score than those of the AK and HAK. In general, AI segmentation algorithms have largely been trained to analyze healthy skin, and so atypia scores should be correlated with visual examination of the lesion and LC‐OCT images. Although standard cutoffs have yet to be established to differentiate normal versus pathologic lesions, atypia scores are valuable in the interim to aid in monitoring treatment response over time and flagging lesions that may warrant biopsy, particularly when interpreted alongside clinical and dermoscopic findings, and when compared to healthy skin.

Ultimately, this study presents a proof‐of‐concept analysis based on three lesions. While the small sample size limits generalizability, these results demonstrate that the DLM can serve as a clinical asset for the non‐invasive detection of atypia, potentially reducing the burden of biopsy [2]. Furthermore, the segmentation time of the DLM is about 3 min, which can benefit dermatologists in clinical practice [2].

Consequently, the use of DLM to analyze LC‐OCT images of pathologic samples for atypia shows great promise, with the potential for rapid and highly sensitive detection of keratinocytic abnormalities, ultimately assisting clinicians in early diagnosis and treatment planning.

## Consent

Written and verbal consent was obtained from participating patients.

## Conflicts of Interest

The authors declare no conflicts of interest.

## IRB

Advara, Pro00035376

## Data Availability

Data available on request from the authors.
